# Knowledge of and Preferences for Medical Aid in Dying

**DOI:** 10.1001/jamanetworkopen.2024.61495

**Published:** 2025-02-24

**Authors:** Elissa Kozlov, Elizabeth A. Luth, Sam Nemeth, Todd D. Becker, Paul R. Duberstein

**Affiliations:** 1 Department of Health Behavior, Society, and Policy, Rutgers School of Public Health, Piscataway, New Jersey; 2 Department of Family Medicine and Community Health, Rutgers University, New Brunswick, New Jersey; 3 Division of Public Health Sciences, Department of Surgery, Washington University School of Medicine in St Louis, St Louis, Missouri

## Abstract

**Importance:**

Medical aid in dying (MAID) is legal in jurisdictions covering more than one-fifth of the US population and has been used by a largely White, educated population. The extent to which knowledge of MAID and preferences for personal use align with patterns of use is unknown.

**Objective:**

To evaluate knowledge among US adults of the legal status of MAID as well as interest in using MAID.

**Design, Setting, and Participants:**

This cross-sectional, self-report online survey study used a national Prime Panels–based sample of US adults recruited between July 16 and August 10, 2024. A total of 3227 respondents aged 18 years or older from all US states and Washington, DC, were included. Individuals from states with legal MAID, aged 60 years or older, and members of racial and ethnic minority groups were oversampled.

**Main Outcomes and Measures:**

Knowledge of the legality of MAID in the US and in respondents’ state of residence as well as potential interest in future use of MAID. Descriptive statistics and χ^2^ tests were used to examine associations by demographic characteristics and place of residence.

**Results:**

Of 3227 respondents (mean [SD] age, 55.7 [17.4] years; 1839 women [57.0%]), 1654 (51.3%) did not know if MAID was legal the US, and 1638 (50.8%) did not know if MAID was legal in their state. Respondents in states where MAID is legal were more likely to know that MAID is legal in the US compared with those in states where MAID is not legal (728 of 2164 [33.6%] vs 216 of 1063 [20.3%]; *P* < .001). Across all respondents, 1420 (44.0%), including 96 of 222 Asian respondents (43.2%), 206 of 605 Black respondents (34.0%), and 129 of 308 Hispanic respondents (41.9%), reported they would definitely or probably consider MAID if they received a diagnosis of a terminal illness.

**Conclusions and Relevance:**

In this online survey study of US adults, substantial interest in using MAID as well as significant knowledge gaps regarding the legality of MAID were reported. These findings highlight the need for public education, policy initiatives, and patient-clinician discussions to ensure equitable access to patient-centered end-of-life options and informed decision-making.

## Key Points

**Question:**

What is the level of public knowledge about the legality of medical aid in dying (MAID) in the US?

**Findings:**

In this online survey study of 3227 US adults, 51.3% did not know if MAID was legal in the US, and 50.8% did not know if MAID was legal in their state. In the full sample, 44.0% expressed interest in using MAID if terminally ill.

**Meaning:**

This study suggests that there is substantial interest across all demographic groups in using MAID, but significant knowledge gaps exist about its legality, which may be associated with observed racial and ethnic and educational differences in MAID’s use.

## Teaser

This survey study evaluates knowledge among US adults of the legal status of medical aid in dying as well as personal interest in using medical aid in dying.

## Introduction

In the US, approximately 74 million people (22%) live in a jurisdiction that allows medical aid in dying (MAID),^1^ a legal practice that allows terminally ill patients to obtain a prescription for medication to end their life. MAID is legal in Washington, DC, and 10 US states (Oregon, Washington, California, Hawaii, Colorado, New Mexico, New Jersey, Vermont, Maine, and Montana, which legalized MAID through Supreme Court ruling) with an additional 3 states (Michigan, New York, and Pennsylvania) considering similar laws.^2^ Despite the high level of public acceptance for MAID—Gallup polls for more than 3 decades reveal most people in the US support MAID^3^—and the increasing legal availability of MAID, there remains limited understanding of the public’s awareness regarding these laws or potential preferences in accessing MAID in the context of terminal illness.

Most patients requesting MAID in the US are non-Hispanic and White (88%-95%), have at least some college education (65%-72%), are aged 60 years or older (74%), and have a primary diagnosis of cancer (69%-74%).^4^ Given that 89% of individuals who die by MAID are enrolled in hospice,^4^ that MAID laws have been in effect the longest in states with predominantly White populations (eg, Oregon),^5^ and that, historically, hospice enrollees tend to be disproportionately White, older, and have cancer,^6^ the profile of the typical MAID user is not surprising. However, MAID laws have recently passed in locations that are more racially and ethnically diverse, (eg, California, Hawaii, New Jersey, and Washington, DC),^5^ and growing proportions of hospice enrollees have primary diagnoses other than cancer (eg, neurologic diseases, heart failure).^6^

Given the trend toward MAID laws being available to an increasingly diverse patient population, research is needed to elucidate if observed demographic differences in MAID use^4^ reflect personal preferences or are the result of access barriers such as knowledge gaps. This study aimed to describe the knowledge of MAID’s legality and potential personal preferences about using MAID among US adults living in MAID-legal states (MLS), including Washington, DC, as well as in non–MAID-legal states (nMLS).

## Methods

### Study Design

All data for this cross-sectional study were collected via a participant self-administered online survey using Qualtrics. The Rutgers University institutional review board approved this study and deemed it to be exempt from human participants review due to minimal risk to participants and the complete deidentification of data. Inclusion criteria comprised age 18 years or older, residency in the US, proficiency in reading English, and internet access. Eligible participants who completed the survey were compensated through CloudResearch using a variable and decentralized compensation strategy. Participants agreed to a compensation amount from CloudResearch prior to consenting to the survey. Informed consent was obtained from all participants on the first page of the web-based survey. This study followed the American Association for Public Opinion Research (AAPOR) reporting guideline.

### Participant Recruitment

A national Prime Panels–based sample of participants was recruited between July 16 and August 10, 2024, using CloudResearch. CloudResearch uses Prime Panels by aggregating respondents from a network of 225 vetted sources such as survey panels, loyalty programs, and mobile apps to reach a diverse pool of respondents. Prime Panels’ structured targeting and quality measures create a more reliable and representative option compared with pure convenience sampling.^7^ Participants were notified of our study by using generic external survey names to avoid disclosing information about the survey content.

We oversampled respondents from MLS, respondents aged 60 years or older, and respondents from members of racial and ethnic minority groups to facilitate analyses. Of 3834 total responses, 3371 individuals met criteria for inclusion in the analytic sample: completed 5 questions on MAID legality and attitudes (n = 3509), indicated state of residence (n = 3487), aged 18 years or older (n = 3478), identified as male or female (n = 3459), and took at least 1 minute to complete the survey (n = 3371). Less than 5% of the overall sample (4.3%) were missing data on any covariate. Additionally, less than 1% of each covariate was missing data. As such, we analyzed data on 3227 individuals with complete information on all variables.

### Measures

#### Demographics

Participants self-reported all survey data including demographic items assessing age, state of residence (including Washington, DC), educational level, sex, race (Asian, Black or African American, White, other race, or >1 race), and Hispanic ethnicity. Other race was composed of groups with small cell sizes and included American Indian or Alaska Native, Middle Eastern or North African, and Native Hawaiian or Pacific Islander. Racial and ethnic categories were taken from the US Census. The study assessed racial and ethnic variables to help elucidate reasons behind the marked differences in MAID use across racial and ethnic groups reported in prior literature. The survey also asked about religious affiliation, frequency of participation in religious events, political tendencies, and financial stability.

#### MAID Knowledge and Preferences for Future Use

MAID was defined for participants as, “laws that permit physicians to assist in ending an individual’s life by prescribing medicines that are intended to cause death. In some countries, MAID is legal and available to patients who have less than 6 months to live due to a serious illness.” Participants were asked if MAID is legal anywhere in the US and if MAID is legal in their state of residence (yes, no, or do not know). Participants were then informed that MAID is legal in some states in the US for patients with 6 months or fewer to live due to a serious illness. Participants were asked if they would personally consider pursuing MAID “if diagnosed with a terminal illness that would certainly cause death within 6 months” (definitely, probably, not sure, probably not, or definitely not).

### Statistical Analysis

We calculated descriptive statistics for the sample and in MLS strata (yes or no). We used χ^2^ tests to evaluate associations between demographic characteristics and MLS strata with knowledge of MAID legality and preferences for MAID use. We also conducted a multinomial logistic regression of individuals in MLS to evaluate the association between the maturity of MAID laws and correctly or incorrectly identifying if MAID is legal in the state. Analysis was performed with StataMP, version 18.0 (Stata Corp). All *P* values were from 2-sided tests, and results were deemed statistically significant at *P* < .05.

## Results

### Sample Demographics

Descriptive statistics are presented in Table. The 3227 survey respondents had a mean (SD) age of 55.7 (17.4) years; 1839 (57.0%) were female and 1388 (43.0%) were male; 222 (6.9%) were Asian, 605 (18.8%) were Black, 308 (9.5%) were Hispanic, and 2100 (65.1%) were White. Nearly three-quarters (2350 [72.8%]) had some college education or more. Two-thirds (2164 [67.1%]) lived in an MLS.

**Table.** Descriptive Statistics for Respondents to Online Survey

Characteristic	No. (%)			*P* value
	Total sample (N = 3227 [100%])	Legality of MAID in respondent’s state		
		Not legal (n = 1063 [33%])	Legal (n = 2164 [67%])	
MAID knowledge				
Legal in US				
No	629 (19.5)	272 (25.6)	357 (16.5)	<.001
Yes	944 (29.3)	216 (20.3)	728 (33.6)	
Do not know	1654 (51.3)	575 (54.1)	1079 (49.9)	
Respondent accurately identifies state legality				
No	685 (21.2)	83 (7.8)	602 (27.8)	<.001
Yes	904 (28.0)	455 (42.8)	449 (20.8)	
Do not know	1638 (50.8)	525 (49.4)	1113 (51.4)	
MAID attitudes				
Should be legal				
No	585 (18.1)	229 (21.5)	356 (16.5)	<.001
Yes	1804 (55.9)	530 (49.9)	1274 (58.9)	
Not sure	838 (26.0)	304 (28.6)	534 (24.7)	
Moral				
No	656 (20.3)	282 (26.5)	374 (17.3)	<.001
Yes	1929 (59.8)	551 (51.8)	1378 (63.7)	
Do not know	642 (19.9)	230 (21.6)	412 (19.0)	
Would use MAID				
Definitely not	414 (12.8)	165 (15.5)	249 (11.5)	<.001
Probably not	402 (12.5)	158 (14.9)	244 (11.3)	
Not sure	991 (30.7)	327 (30.8)	664 (30.7)	
Probably	763 (23.6)	216 (20.3)	547 (25.3)	
Definitely	657 (20.4)	197 (18.5)	460 (21.3)	
Demographics				
Age [range, 18-98], mean (SD), y	55.7 (17.4)	54.9 (16.9)	56.1 (17.7)	.10
Sex				
Male	1388 (43.0)	477 (44.9)	911 (42.1)	.14
Female	1839 (57.0)	586 (55.1)	1253 (57.9)	
Race				
Asian	222 (6.9)	49 (4.6)	173 (8.0)	<.001
Black	605 (18.8)	336 (31.6)	269 (12.4)	
White	2100 (65.1)	578 (54.4)	1522 (70.3)	
Other or >1 race^a^	300 (9.3)	100 (9.4)	200 (9.2)	
Hispanic				
No	2919 (90.5)	971 (91.4)	1948 (90.0)	.23
Yes	308 (9.5)	92 (8.7)	216 (10.0)	
Educational level				
High school or less	877 (27.2)	332 (31.2)	545 (25.2)	.003
Some college	1147 (35.5)	365 (34.3)	782 (36.1)	
Bachelor’s degree	787 (24.4)	244 (23.0)	543 (25.1)	
Graduate degree	416 (12.9)	122 (11.5)	294 (13.6)	
Religion				
Atheist	188 (5.8)	44 (4.1)	144 (6.7)	<.001
Agnostic	233 (7.2)	62 (5.8)	171 (7.9)	
Baptist	346 (10.7)	204 (19.2)	142 (6.6)	
Catholic	682 (21.1)	205 (19.3)	477 (22.0)	
Jewish	81 (2.5)	18 (1.7)	63 (2.9)	
Protestant	604 (18.7)	189 (17.8)	415 (19.2)	
Other	861 (26.7)	275 (25.9)	586 (27.1)	
Prefer not to answer	232 (7.2)	66 (6.2)	166 (7.7)	
Religious frequency				
Never	1046 (32.4)	287 (27.0)	759 (35.1)	<.001
Seldom	1149 (35.6)	346 (32.6)	803 (37.1)	
Regularly	624 (19.3)	261 (24.6)	363 (16.8)	
Often	408 (12.6)	169 (15.9)	239 (11.0)	
Financial well-being				
Not enough to make ends meet	675 (20.9)	235 (22.1)	440 (20.3)	.38
Enough to make ends meet	1572 (48.7)	519 (48.8)	1053 (48.7)	
Comfortable	980 (30.4)	309 (29.1)	671 (31.0)	
Political leaning				
Very conservative	351 (10.9)	116 (10.9)	235 (10.9)	.10
Somewhat conservative	628 (19.5)	211 (19.9)	417 (19.3)	
Neither liberal nor conservative	1156 (35.8)	398 (37.4)	758 (35.0)	
Liberal	692 (21.4)	198 (18.6)	494 (22.8)	
Very liberal	400 (12.4)	140 (13.2)	260 (12.0)	

Abbreviation: MAID, medical aid in dying.

^a^ Other race includes American Indian or Alaska Native, Middle Eastern or North African, and Native Hawaiian or Pacific Islander.

### MAID Knowledge

#### Overall Sample

In the overall sample (Table), over half of respondents (1654 [51.3%]) did not know if MAID was legal anywhere in the US, 629 (19.5%) incorrectly indicated it was not legal anywhere in the US, and 944 (29.3%) knew it was legal somewhere in the US. Respondents from MLS more often identified MAID as legal in the US than those from nMLS (728 of 2164 [33.6%] vs 216 of 1063 [20.3%]). Uncertainty about MAID legality was high in both groups: 1079 of 2164 MLS respondents (49.9%) and 575 of 1063 nMLS respondents (54.1%) indicated they did not know if MAID was legal in the US. Respondents in MLS indicated MAID was not legal in the US less often than expected (357 of 2164 [16.5%]), while those from nMLS indicated MAID was not legal more often than expected (272 of 1063 [25.6%]; χ^2^_2_ = 76.0; *P* < .001).

Most respondents (1638 [50.8%]) did not know if MAID was legal where they lived, 685 (21.2%) incorrectly identified its legality in their location, and 904 (28.0%) correctly identified whether MAID was legal in their state of residence (including Washington, DC) (Table). Relatively equal percentages of respondents in MLS and nMLS did not know if MAID was legal where they lived (1113 of 2164 [51.4%] and 525 of 1063 [49.4%], respectively). Respondents from MLS were incorrect about the status of MAID in their state more often than expected (602 of 2164 [27.8%]), compared with only 83 of 1063 respondents living in nMLS (7.8%) (χ^2^_2_ = 258.8; *P* < .001).

#### Knowledge by State of Residence

**Figure 1.**
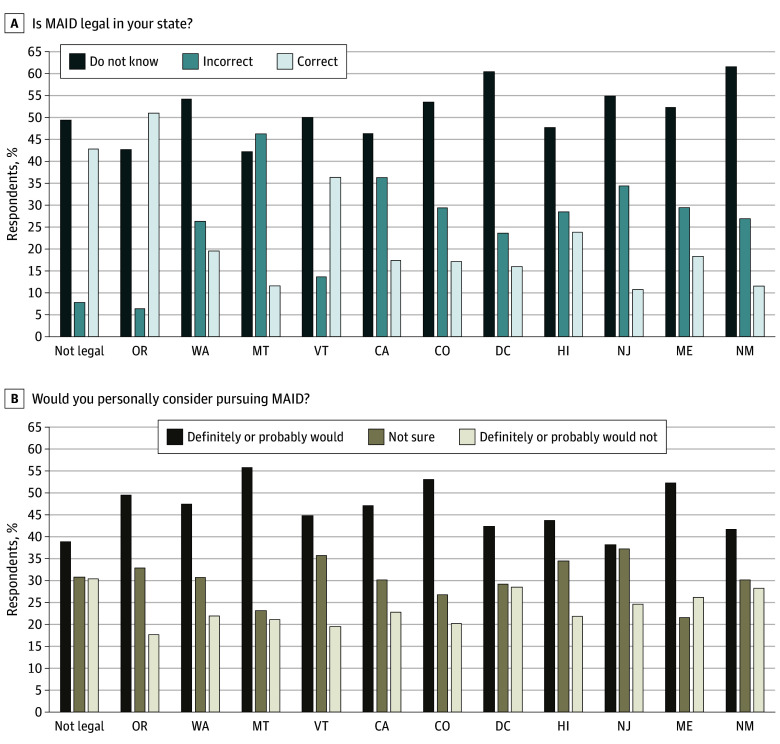
Percentage of Respondents Correctly Indicating Whether Medical Aid in Dying (MAID) Is Legal in Their State and Whether They Would Personally Consider Using MAID If They Received a Diagnosis of a Terminal Illness States are listed in chronological order of when they became legal, starting first in Oregon (1998) and most recently in New Mexico (2021). Respondents were asked whether MAID is legal in the state in which they lived and were coded as responding “do not know” or correctly or incorrectly answering the question. Respondents were asked whether they would personally consider using MAID if they received a diagnosis of a terminal illness that would certainly cause death within 6 months. Not legal comprises all respondents from states where MAID is not legal.

Considering respondents in every MLS and nMLS, more than half of respondents indicated (range, 52.0% in Oregon [106.8 of 204] to 80% in nMLS [850.4 of 1063]) that they did not know or answered incorrectly that MAID was legal in the US (eTable 1 in Supplement 1). At least half of respondents in all MLS, except for Oregon, did not know or answered incorrectly that MAID was legal in their state (Figure 1). Between 10.7% and 51.0% of respondents in MLS correctly identified MAID as legal in their state (10.7% [34 of 317] in New Jersey, where MAID has been legal since 2019; and 51.0% [104 of 204] in Oregon, where MAID has been legal since 1998). Results of a multinomial logistic regression model revealed that there was a slight improvement in correct answers among residents of MLS as MAID laws mature (yes, correct: relative risk ratio [RRR], 1.06 [SE, 0.01; 95% CI, 1.05-1.07]; *P* < .001; no, incorrect: RRR, 0.98 [SE, 0.01; 95% CI, 0.96-0.99]; *P* < .010; reference group = did not know). A substantial minority (455 of 1063 [42.8%]) of respondents in nMLS correctly identified that MAID was not legal in their state.

#### Knowledge by Demographic Factors

**Figure 2.**
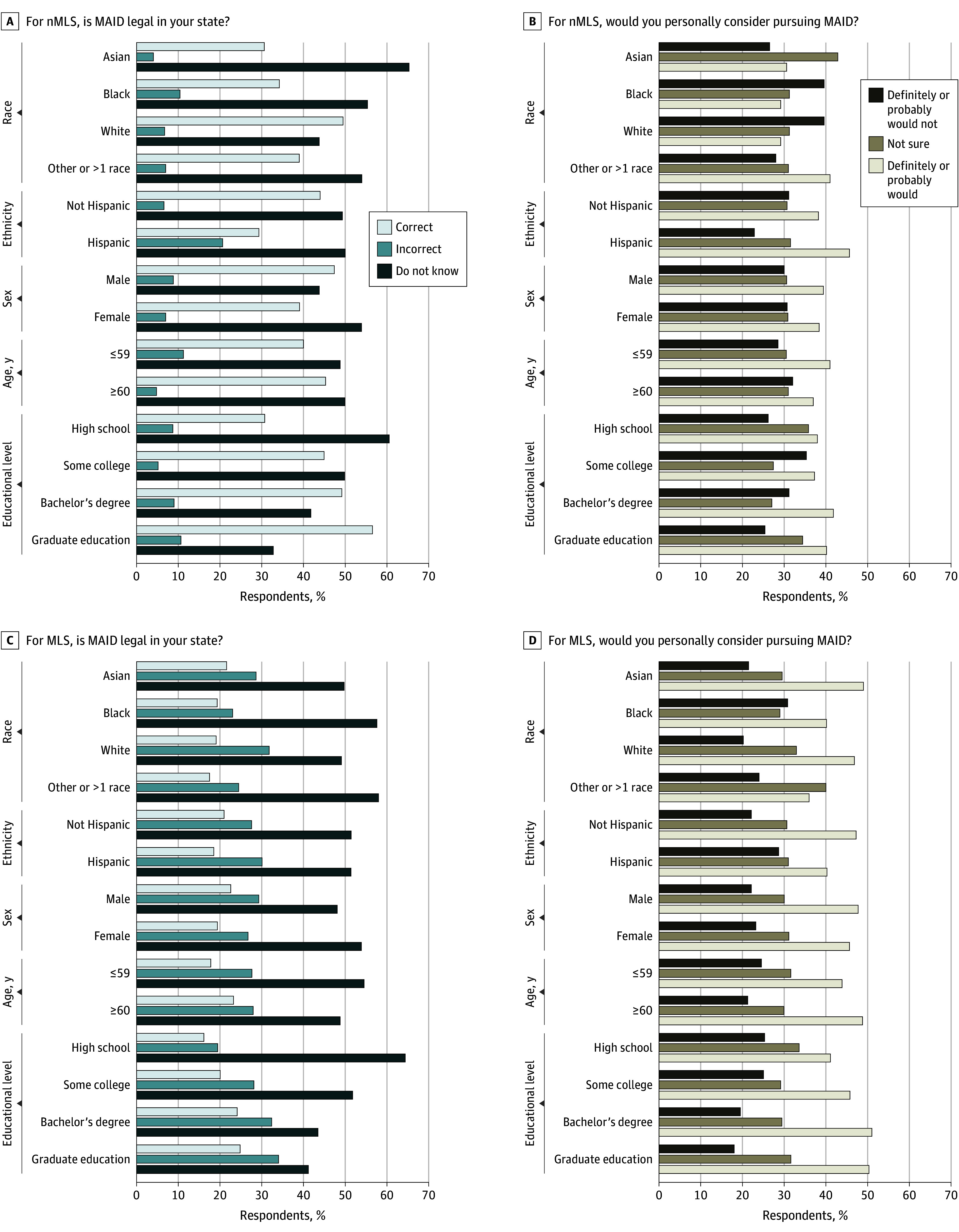
Participant Responses to Select Survey Questions According to Demographic Groups Respondents were asked whether they would personally consider using medical aid in dying (MAID) if they received a diagnosis of a terminal illness that would certainly cause death within 6 months. MAID-legal states (MLS) include California, Colorado, Hawaii, Maine, Montana, New Jersey, New Mexico, Oregon, Vermont, Washington, and Washington, DC. Other race includes American Indian or Alaska Native, Middle Eastern or North African, and Native Hawaiian or Pacific Islander. nMLS indicates non–MAID-legal states.

Figure 2 graphs proportions of respondents by demographic characteristics and whether they resided in MLS or nMLS who responded do not know, no, and yes to the question if MAID was legal anywhere in the state in which they lived (including Washington, DC). With few exceptions, do not know constituted the largest proportion of responses regardless of respondent race, Hispanic ethnicity, educational level, sex, age, or religion in both MLS and nMLS. Full results are provided in eTables 1 to 6 in Supplement 1.

### Consideration of Personal MAID Use

#### Overall Sample

As reported in the Table, in the overall sample, 1420 (44.0%) of respondents definitely or probably would consider using MAID if they received a diagnosis of a terminal illness that would certainly cause death within 6 months, 991 (30.7%) were not sure, and 816 (25.3%) of respondents would definitely or probably not consider using MAID. Among respondents from MLS, 1007 of 2164 (46.5%) would definitely or probably consider using MAID, 664 of 2164 (30.7%) were not sure, and 493 of 2164 (22.8%) would definitely or probably not consider using it. Among respondents from nMLS, 413 of 1063 (38.9%) would definitely or probably consider using MAID, 327 of 1063 (30.8%) were not sure, and 323 of 1063 (30.4%) would definitely or probably not consider using MAID. Overall, respondents in MLS indicated they definitely or probably would not use MAID less often than expected (493 of 2164 [22.8%]); those from nMLS chose these response categories more often than expected (323 of 1063 [30.4%]; χ^2^_4_ = 26.3; *P* < .001).

#### Preference by State of Residence

Figure 1 graphs responses about consideration of personal preferences for MAID by MLS state of residence (nMLS are a single group). In all states, including nMLS, the largest proportion of respondents indicated that they definitely or probably would consider using MAID if they received a diagnosis of a terminal illness. In nearly all states, respondents who definitely or probably would not consider using MAID represented the smallest group (range, 17.6% in Oregon [36 of 204] to 30.4% in nMLS [323 of 1063]). Maine was the only exception to this trend, where 21.6% (33 of 153) were not sure and 26.1% (40 of 153) definitely or probably would not use MAID.

#### Preference by Demographic Factors

Figure 2 graphs proportions of respondents who would consider using MAID, are not sure, and would not consider it by demographic characteristics and whether they resided in MLS or nMLS. With few exceptions (eg, Baptist, Black, and Asian respondents in nMLS), probably or definitely would consider using MAID constituted the largest proportion of responses regardless of respondent sex, age, religion, or Hispanic ethnicity. In our sample, 350 of 877 respondents with a high school education or less (39.9%) would definitely or probably consider using MAID if they had a terminal diagnosis. A total of 96 of 222 Asian respondents (43.2%), 206 of 605 Black respondents (34.0%), and 129 of 308 Hispanic respondents (41.9%) reported they would definitely or probably consider MAID if they received a diagnosis of a terminal illness. Black respondents, Baptist respondents, and those of other religions from nMLS selected that they would definitely or probably not consider using MAID more often than expected (133 of 336 [39.6%], 77 of 204 [37.7%], and 102 of 275 [37.1%], respectively); as did Baptist respondents from MLS (50 of 142 [35.2%]) (nMLS race, χ^2^_6_ = 30.9; n = 1063; nMLS religion, χ^2^_14_ = 55.3; n = 1063; and MLS religion, χ^2^_14_ = 119.2; n = 2164; *P* < .001). Asian respondents from nMLS and those of other or more than 1 race from MLS indicated they were not sure if they would consider using MAID more often than expected (21 of 49 [42.9%] and 80 of 200 [40.0%], respectively) (race nMLS, χ^2^_6_ = 30.9; n = 1063; race MLS, χ^2^_6_ = 25.5; n = 2164; *P* < .001). Further results are provided in eTables 1 to 6 in Supplement 1.

## Discussion

Recognizing the growing number of requests as well as the lack of research on MAID, organizations^8,9^ have called for formative research on the topic. Findings from this national study highlight a considerable level of interest in potentially using MAID and a general lack of public knowledge about MAID’s legal status. Approximately half of the respondents in our sample were unsure whether MAID was legal anywhere in the US or in their own state of residence. Given the relatively high levels of education in our sample,^10^ this finding is likely an underestimate of the true prevalence of uncertainty. To reduce barriers to all types of end-of-life care, enhanced public education about end-of-life options, including advanced care planning, palliative care, hospice, home care, and MAID, is needed to ensure that individuals are adequately informed about all their care options at end of life. Although opponents of MAID have raised concerns about potential overuse of MAID, there has been no evidence of widespread MAID abuse in the US since MAID was legalized in Oregon in 1998.^11^

The results of this study also challenge several common perceptions about preferences toward using MAID. Although prior studies show that individuals who use MAID are overwhelmingly White and have at least some college education,^4^ our findings indicate that interest in using MAID spans a broad range of racial and ethnic and educational strata. In our sample, 39.9% of respondents with a high school education or less would definitely or probably consider using MAID if they had a terminal diagnosis. The same is true of 43% of Asian respondents, 34% of Black respondents, and 42% of Hispanic respondents. Given that these groups are almost nonexistent in MAID use reports,^4^ our findings suggest that systemic barriers—financial constraints, health literacy gaps, insurance and reimbursement concerns, practitioner bias, and difficulties navigating the health care system—may limit access to MAID among individuals with lower educational levels and members of racial and ethnic minority groups. Moreover, our study aligns with international research from countries such as Switzerland, the Netherlands, and Canada, where interest in using MAID is high, but MAID requests are also concentrated among older patients with higher socioeconomic status.^12,13,14,15,16,17^ These patterns raise critical questions about equity in access to MAID.

The role of hospice^18,19^ may help explain the demographic discrepancies between those who express interest in MAID and those who ultimately use it. Prior studies have found that up to 87% of individuals who die by MAID had been enrolled in hospice,^4^ suggesting that hospice is associated with MAID use, although little is known about how hospices in MLS consider MAID in their clinical practice. The association between MAID and hospice has been variable; some major organizations have opposed MAID, while others have taken positions of neutrality.^20,21,22^ Hospice care in the US has historically had fewer enrollees from racial and ethnic minority groups,^6^ which could limit exposure to information about MAID for these groups. Furthermore, although primary diagnoses in hospice have shifted over time,^6^ hospice enrollees tend to be older adults with cancer^6^—a profile that mirrors the typical MAID user.^4^ Individuals who do not fit this profile in MLS may be less likely to be aware of MAID.

Knowledge of MAID’s legal status was very low, even in states with established MAID laws, which is likely associated with discrepancies in MAID’s use patterns among members of racial and ethnic minority groups and individuals with less education. This lack of awareness could stem from limited dissemination of information about MAID’s availability, lack of resources devoted to dissemination efforts, cultural taboos and shame surrounding end-of-life discussions,^23^ low numbers of physicians who are willing to be MAID prescribers, and relatively recent implementation of MAID laws in some states, although MAID law maturity was only slightly associated with improved knowledge in our sample.^24,25^ Improved knowledge about all end-of-life care options, including MAID, for all adults is essential because decisions regarding end-of-life care often involve significant planning and discussions among patients, families, and health care professionals.^26^ Increasing awareness can facilitate earlier advanced care planning, better support for terminally ill relatives, and informed decision-making when end-of-life care becomes relevant.^27,28^

The relatively high levels of openness to MAID observed in our study and in prior research on MAID^29^ may be influenced by several historical and psychological factors. Graphic depictions of heroic but futile efforts to prevent death, such as aggressive chemotherapy administered until the final days of life,^30^ have further heightened public awareness of the emotional and physical toll of such interventions,^23,31^ which may be associated with greater receptivity to MAID. Death anxiety^32^ may drive individuals to seek control over their death, with MAID presenting an avenue for autonomy in the face of impending mortality. Secular shifts in cultural attitudes toward death and dying have led to the creation of death cafés and the emergence of death doulas.^31^ Contemporary debates about MAID are occurring in a cultural context that has changed significantly over the past few decades, one characterized by a shift toward greater openness and acceptance of MAID and an increasingly diverse “death marketplace.”

### Limitations

Several limitations should be considered when interpreting these findings. First, the use of a Prime-Panels–based sample somewhat limits the generalizability of our results, as individuals who participate in online surveys may differ systematically from the broader population. As noted, our sample likely overestimated knowledge of MAID legality and potentially overestimated preferences for using MAID in the future. In addition, current health status of the participants was unknown, and health status may be an important predictor of knowledge of end-of-life care options. Second, the cross-sectional design prevents any conclusions about causality. Third, unmeasured factors (eg, personal experiences with end-of-life care) may influence awareness of and preferences toward MAID. Fourth, surveyed preferences for treatments or services in hypothetical scenarios are imperfect predictors of preferences or use in clinical situations. Fifth, all self-reported data on sensitive topics are subject to social desirability biases.

## Conclusions

This survey study of adults living in the US underscored a significant lack of public knowledge regarding the legality of MAID across the US, even among individuals living in jurisdictions where it is legally available. These findings added novel insights into the broader discourse on MAID by highlighting the disparity between expressed interest in potentially using MAID, particularly among persons from racial and ethnic minority groups and with lower levels of education, and actual MAID use patterns in the US and internationally. Our findings indicate a need for enhanced public education about the legality of MAID to ensure individuals are informed about end-of-life care options in MLS. In addition, addressing the informational and structural barriers that potentially prevent members of racial and ethnic minority groups and those with lower levels of education from accessing MAID is an important component of equitable end-of-life care. Future research should focus on understanding the reasons, including psychological factors associated with favorable attitudes about MAID, for these knowledge gaps to promote informed decision-making for all individuals at the end of life.

## Notes

Supplement 1. **eTable 1.** Percentage of Responses to Questions About Knowledge of MAID Legality in the US and State of Residence for 3,227 Online Survey Respondents **eTable 2.** Percentages of Responses to the Question “Would You Personally Consider Pursuing Medical Aid in Dying If You Were Diagnosed With a Terminal Illness That Would Certainly Cause Death Within 6 Months?” for 3,227 Online Survey Respondents **eTable 3.** Percentages of Responses to Questions About Knowledge of MAID Legality in the US and Their State of Residence for 1,063 Online Survey Respondents Residing in States Where MAID Is Not Legal **eTable 4.** Percentages of Responses to Questions About Knowledge of MAID Legality in the US and Their State of Residence for 2,165 Online Survey Respondents Residing in States Where MAID Is Legal **eTable 5.** Percentages of Responses to the Question “Would You Personally Consider Pursuing Medical Aid in Dying If You Were Diagnosed With a Terminal Illness That Would Certainly Cause Death Within 6 Months?” for 1,063 Online Survey Respondents Residing in States Where MAID Is Not Legal **eTable 6.** Percentages of Responses to the Question “Would You Personally Consider Pursuing Medical Aid in Dying If You Were Diagnosed With a Terminal Illness That Would Certainly Cause Death Within 6 Months?” for 2,165 Online Survey Respondents Residing in States Where MAID Is Legal (Including Washington, DC)

Supplement 2.
Data Sharing Statement
