# Supplementary material for: Knowledge of and Preferences for Medical Aid in Dying
Source: JAMA Netw Open. 2025 Feb 24;8(2):e2461495. doi: 10.1001/jamanetworkopen.2024.61495 (PMC11851238; doi:10.1001/jamanetworkopen.2024.61495)
Supplement: Supplement 1. — eTable 1. Percentage of Responses to Questions About Knowledge of MAID Legality in the US and State of Residence for 3,227 Online Survey Respondents eTable 2. Percentages of Responses to the Question “Would You Personally Consider Pursuing Medical Aid in Dying If You Were Diagnosed With a Terminal Illness That Would Certainly Cause Death Within 6 Months?” for 3,227 Online Survey Respondents eTable 3. Percentages of Responses to Questions About Knowledge of MAID Legality in the US and Their State of Residence for 1,063 Online Survey Respondents Residing in States Where MAID Is Not Legal eTable 4. Percentages of Responses to Questions About Knowledge of MAID Legality in the US and Their State of Residence for 2,165 Online Survey Respondents Residing in States Where MAID Is Legal eTable 5. Percentages of Responses to the Question “Would You Personally Consider Pursuing Medical Aid in Dying If You Were Diagnosed With a Terminal Illness That Would Certainly Cause Death Within 6 Months?” for 1,063 Online Survey Respondents Residing in States Where MAID Is Not Legal eTable 6. Percentages of Responses to the Question “Would You Personally Consider Pursuing Medical Aid in Dying If You Were Diagnosed With a Terminal Illness That Would Certainly Cause Death Within 6 Months?” for 2,165 Online Survey Respondents Residing in States Where MAID Is Legal (Including Washington, DC) [file jamanetwopen-e2461495-s001.pdf]

Supplementary Online Content

Kozlov E, Luth EA, Nemeth S, Becker TD, Duberstein PR. Knowledge of and preferences for medical aid in dying in the US. *JAMA Netw Open.* 2025;8(2):e2461495. doi:10.1001/jamanetworkopen.2024.61495

eTable 1. Percentage of Responses to Questions About Knowledge of MAID Legality in the US and State of Residence for 3,227 Online Survey Respondents

eTable 2. Percentages of Responses to the Question "Would You Personally Consider Pursuing Medical Aid in Dying if You Were Diagnosed With a Terminal Illness That Would Certainly Cause Death Within 6 Months?" for 3,227 Online Survey Respondents

eTable 3. Percentages of Responses to Questions About Knowledge of MAID Legality in the US and Their State of Residence for 1,063 Online Survey Respondents Residing in States Where MAID is Not Legal

eTable 4. Percentages of Responses to Questions About Knowledge of MAID Legality in the US and Their State of Residence for 2,165 Online Survey Respondents Residing in States Where MAID is Legal

eTable 5. Percentages of Responses to the Question "Would You Personally Consider Pursuing Medical Aid in Dying if You Were Diagnosed With a Terminal Illness That Would Certainly Cause Death Within 6 Months?" for 1,063 Online Survey Respondents Residing in States Where MAID is Not Legal

eTable 6. Percentages of Responses to the Question "Would You Personally Consider Pursuing Medical Aid in Dying if You Were Diagnosed With a Terminal Illness That Would Certainly Cause Death Within 6 Months?" for 2,165 Online Survey Respondents Residing in States Where MAID is Legal (Including Washington, DC)

This supplementary material has been provided by the authors to give readers additional information about their work.

© 2025 Kozlov E et al. *JAMA Network Open.*

eTable 1. Percentage of Responses to Questions About Knowledge of MAID Legality in the US and State of Residence for 3,227 Online Survey Respondents

	**Is MAID legal in the US?**			**Is MAID legal in the state in which you live?**		
	Don't know	*No*	*Yes*	*Don't know*	*Incorrectly answered*	*Correctly answered*
Non-MAID legal states	54%	26%	20%	49%	8%	43%
California	51%	20%	29%	46%	36%	17%
Colorado	46%	18%	36%	54%	29%	17%
Hawaii	50%	17%	34%	48%	28%	24%
Maine	48%	16%	35%	52%	29%	18%
Montana	52%	20%	28%	42%	46%	12%
New Jersey	59%	17%	24%	55%	34%	11%
New Mexico	55%	17%	28%	62%	27%	12%
Oregon	38%	14%	48%	43%	6%	51%
Vermont	44%	11%	45%	50%	14%	36%
Washington	45%	15%	40%	54%	26%	20%
Washington, D.C.	60%	14%	26%	60%	24%	16%

© 2025 Kozlov E et al. *JAMA Network Open.*

eTable 2. Percentages of Responses to the Question “Would You Personally Consider Pursuing Medical Aid in Dying if You Were Diagnosed With a Terminal Illness That Would Certainly Cause Death Within 6 Months?” for 3,227 Online Survey Respondents

	**Definitely/ Probably would**	**Not sure**	**Definitely/ Probably would not**
Non-MAID legal states	39%	31%	30%
California	47%	30%	23%
Colorado	53%	27%	20%
Hawaii	44%	34%	22%
Maine	52%	22%	26%
Montana	56%	23%	21%
New Jersey	38%	37%	25%
New Mexico	42%	30%	28%
Oregon	50%	33%	18%
Vermont	45%	36%	19%
Washington	47%	31%	22%
Washington, D.C.	42%	29%	28%

© 2025 Kozlov E et al. *JAMA Network Open.*

eTable 3. Percentages of Responses to Questions About Knowledge of MAID Legality in the US and Their State of Residence for 1,063 Online Survey Respondents Residing in States Where MAID is Not Legal

	Is MAID legal in the US?				Is MAID legal in the state in which you live?			
	Don't know	*No*	*Yes*	*p value*	*Don't know*	*Incorrectly answered*	*Correctly answered*	*p value*
**Race**				0.10				<0.001
White	51%	28%	21%		44%	7%	49%	
Black	57%	24%	19%		55%	10%	34%	
Asian	63%	29%	8%		65%	4%	31%	
Other/>1 Race	54%	20%	26%		54%	7%	39%	
**Ethnicity**				0.02				<0.001
Not Hispanic/ Latino	54%	26%	19%		49%	7%	44%	
Hispanic/ Latino	52%	17%	30%		50%	21%	29%	
**Education**				0.02				<0.001
High school or less	61%	20%	19%		61%	9%	31%	
Some college	54%	28%	18%		50%	5%	45%	
Bachelor's degree	50%	28%	23%		42%	9%	49%	
Graduate degree	47%	26%	27%		33%	11%	57%	
**Sex**				0.013				0.005
Male	50%	30%	20%		44%	9%	47%	
Female	58%	22%	20%		54%	7%	39%	
**Age**				0.07				<0.001
59 or younger	50%	27%	22%		49%	11%	40%	
60 or older	57%	24%	18%		50%	5%	45%	
**Religion**				<0.001				0.01
Atheist	66%	25%	9%		52%	5%	43%	
Agnostic	48%	24%	27%		40%	3%	56%	
Catholic	50%	29%	21%		42%	10%	48%	
Jewish	44%	6%	50%		50%	17%	33%	
Protestant	50%	28%	22%		43%	7%	50%	
Baptist	54%	30%	16%		50%	7%	42%	
Other	59%	21%	20%		57%	8%	35%	
Prefer not to answer	61%	18%	21%		61%	8%	32%	

P value based on chi square tests. Other race=Native Hawaiian/Pacific Islander, Middle Easten/North African, and American Indian/Alaskan Native

© 2025 Kozlov E et al. *JAMA Network Open.*

eTable 4. Percentages of Responses to Questions About Knowledge of MAID Legality in the US and Their State of Residence for 2,165 Online Survey Respondents Residing in States Where MAID is Legal

	**Is MAID legal in the US?**				**Is MAID legal in the state in which you live?**			
	Don't know	*No*	*Yes*	*P value*	*Don't know*	*Incorrectly answered*	*Correctly answered*	*P value*
**Race**				0.002				0.09
White	48%	15%	36%		50%	29%	22%	
Black	55%	19%	26%		58%	23%	19%	
Asian	47%	22%	31%		49%	32%	19%	
Other/>1 Race	58%	17%	26%		58%	25%	18%	
**Ethnicity**				0.07				0.60
Not Hispanic/Latino	50%	16%	34%		51%	28%	21%	
Hispanic/Latino	51%	21%	28%		51%	30%	19%	
**Education**				<0.001				<0.001
High school or less	61%	14%	24%		64%	19%	16%	
Some college	51%	18%	32%		52%	28%	20%	
Bachelor's degree	42%	17%	41%		43%	32%	24%	
Graduate education	40%	17%	43%		41%	34%	25%	
**Sex**				0.07				0.03
Male	47%	18%	35%		48%	29%	23%	
Female	52%	15%	33%		54%	27%	19%	
**Age**				0.01				0.004
59 or younger	54%	18%	29%		55%	28%	18%	
60 or older	47%	15%	38%		49%	28%	23%	
**Religion**				<0.001				<0.001
Atheist	41%	15%	44%		42%	28%	29%	
Agnostic	39%	15%	46%		42%	30%	29%	
Catholic	51%	19%	31%		51%	29%	20%	
Jewish	56%	13%	32%		46%	43%	11%	
Protestant	42%	16%	42%		46%	29%	25%	
Baptist	53%	22%	25%		56%	27%	17%	
Other	55%	16%	29%		56%	26%	18%	
Prefer not to answer	61%	13%	25%		67%	18%	15%	

P value based on chi square tests. Other race=Native Hawaiian/Pacific Islander, Middle Easten/North African, and American Indian/Alaskan Native

© 2025 Kozlov E et al. *JAMA Network Open.*

eTable 5. Percentages of Responses to the Question “Would You Personally Consider Pursuing Medical Aid in Dying if You Were Diagnosed With a Terminal Illness That Would Certainly Cause Death Within 6 Months?” for 1,063 Online Survey Respondents Residing in States Where MAID is Not Legal

	Definitely/ Probably would	Not sure	Definitely/ Probably would not	p value
**Race**				<0.001
White	45%	29%	26%	
Black	29%	31%	40%	
Asian	31%	43%	27%	
Other/>1 Race	41%	31%	28%	
**Ethnicity**				0.21
Not Hispanic/Latino	38%	31%	31%	
Hispanic/Latino	46%	32%	23%	
**Education**				0.050
High school or less	38%	36%	26%	
Some college	37%	27%	35%	
Bachelor's degree	42%	27%	31%	
Graduate education	40%	34%	25%	
**Sex**				0.94
Male	39%	31%	30%	
Female	38%	31%	31%	
**Age**				0.34
59 or younger	41%	31%	29%	
60 or older	37%	31%	32%	
**Religion**				<0.001
Atheist	52%	36%	11%	
Agnostic	52%	34%	15%	
Catholic	46%	25%	29%	
Jewish	78%	22%	0%	
Protestant	38%	31%	31%	
Baptist	29%	33%	38%	
Other	33%	30%	37%	
Prefer not to answer	39%	42%	18%	

P value based on chi square tests. Other race=Native Hawaiian/Pacific Islander, Middle Easten/North African, and American Indian/Alaskan Native

© 2025 Kozlov E et al. *JAMA Network Open.*

eTable 6. Percentages of Responses to the Question "Would You Personally Consider Pursuing Medical Aid in Dying if You Were Diagnosed With a Terminal Illness That Would Certainly Cause Death Within 6 Months?" for 2,165 Online Survey Respondents Residing in States Where MAID is Legal (Including Washington, DC)

	Definitely/ Probably would	Not sure	Definitely/ Probably would not	p value
**Race**				<0.001
White	49%	30%	21%	
Black	40%	29%	31%	
Asian	47%	33%	20%	
Other/>1 Race	36%	40%	24%	
**Ethnicity**				0.06
Not Hispanic/Latino	47%	31%	22%	
Hispanic/Latino	40%	31%	29%	
**Education**				0.006
High school or less	41%	34%	25%	
Some college	46%	29%	25%	
Bachelor's degree	51%	29%	20%	
Graduate education	50%	32%	18%	
**Sex**				0.62
Male	48%	30%	22%	
Female	46%	31%	23%	
**Age**				0.054
59 or younger	44%	32%	25%	
60 or older	49%	30%	21%	
**Religion**				<0.001
Atheist	69%	19%	12%	
Agnostic	70%	23%	7%	
Catholic	43%	33%	24%	
Jewish	48%	37%	16%	
Protestant	40%	30%	29%	
Baptist	31%	34%	35%	
Other	45%	30%	25%	
Prefer not to answer	46%	41%	13%	

P value based on chi square tests. Other race=Native Hawaiian/Pacific Islander, Middle Easten/North African, and American Indian/Alaskan Native

© 2025 Kozlov E et al. *JAMA Network Open.*
