# Supplementary material for: Knowledge of and Preferences for Medical Aid in Dying
Source: JAMA Netw Open. 2025 Feb 24;8(2):e2461495. doi: 10.1001/jamanetworkopen.2024.61495 (PMC11851238; doi:10.1001/jamanetworkopen.2024.61495)
Supplement: Supplement 2. — Data Sharing Statement [file jamanetwopen-e2461495-s002.pdf]

Data Sharing Statement

Kozlov. Knowledge of and Preferences for Medical Aid in Dying in the US. *JAMA Netw Open*. Published February 24, 2025. doi:10.1001/jamanetworkopen.2024.61495

Data
Data available: No

Additional Information
Explanation for why data not available: We have plans to publish additional papers with this data. We will make it available after we have finished analyzing the data.
